# Breastfeeding in primiparous women – expectations and reality: a prospective questionnaire survey

**DOI:** 10.1186/s12884-023-05971-1

**Published:** 2023-09-09

**Authors:** Katrin Oberfichtner, Peter Oppelt, Daniela Fritz, Katharina Hrauda, Christian Fritz, Barbara Schildberger, Julia Lastinger, Patrick Stelzl, Sabine Enengl

**Affiliations:** 1 Department of Gynecology, Obstetrics, and Gynecological Endocrinology, Kepler University Hospital, Johannes Kepler University, Altenbergerstrasse 69, 4040 Linz, Austria; 2 University of Applied Sciences for the Health Professions, Linz, Austria; 3 Institute for Statistical Analysis Jaksch & Partner GmbH, Linz, Austria

**Keywords:** Breastfeeding behaviour, Breastfeeding duration, Expectations, Midwifery care

## Abstract

**Background:**

Breastfeeding provides the optimal nutrition for infants and offers numerous benefits for both mother and child. The World Health Organisation recommends exclusive breastfeeding during the first 6 months of life and the introduction of complementary feeding between the fifth and seventh months of life. There is a discrepancy between breastfeeding recommendations and the actual duration of breastfeeding. The aim of this study was to analyse breastfeeding behaviour in primiparous women in order to be able to provide support for mothers.

**Methods:**

In this prospective, questionnaire-based study conducted between 2020 and 2022, primiparous women were asked to complete three questionnaires at three defined survey time points (routine prepartum presentation, postpartum hospitalization, completed sixth month of life).

**Results:**

A total of 140 women were included and returned all three questionnaires. Fifty-eight percent performed breastfeeding exclusively at least until their baby had reached the age of 6 months, whereas 20% already stopped within the first 6 months. The main reasons given for early cessation were insufficient milk supply and inadequate infant weight gain. A comprehensive level of prepartum knowledge had a significant positive effect on participants’ sense of confidence with breastfeeding. Sociodemographic factors such as age and educational level were also associated with breastfeeding behaviour, but significant corresponding differences in the duration of breastfeeding were not observed. Women with postpartum midwifery care breastfed significantly longer (*p* < 0.05).

**Conclusions:**

Breastfeeding behaviour and duration are influenced by multiple factors. Although certain sociodemographic factors are unalterable, comprehensive prepartum knowledge transfer and postpartum midwifery care have a positive impact on breastfeeding behaviour.

**Trial registration:**

The study was retrospectively registered at the German Clinical Trials Register (*Deutsches Register Klinischer Studien,* DRKS) on 6 December 2022 (DRKS00030763).

**Supplementary Information:**

The online version contains supplementary material available at 10.1186/s12884-023-05971-1.

## Background

Breast milk is considered the gold standard for infant nutrition. The World Health Organisation (WHO) recommends exclusive breastfeeding during the first 6 months of life. After the introduction of complementary feeding, additional breastfeeding should be provided at least until the child reaches 2 years of age [1]. With sufficient milk production and satisfactory breastfeeding technique, most infants’ energy and nutrient needs can be easily met during the first 6 months of life [2]. From 6 months to 1 year of age, breast milk provides 50% of energy and nutrient needs, and about one-third in the second year of life. As the nutrients in breast milk are much more valuable than those in complementary foods, additional breastfeeding should be continued after the introduction of complementary feeding [1].

These recommendations are based on the results of studies showing that exclusive breastfeeding significantly reduces child morbidity and mortality in comparison with partial breastfeeding or non-breastfeeding [3]. Long-term effects of breastfeeding include a reduction in the risk of becoming overweight or developing type 2 diabetes mellitus [4, 5].

The positive effects of breastfeeding on the mother can already be observed immediately postpartum. Breastfeeding promotes the oxytocin-induced uterine contraction that reduces postpartum blood loss [6]. Moreover, oxytocin enhances the development of a healthy mother–infant bond and protects against postpartum depression [7]. Breastfeeding also protects mothers from developing arterial hypertension, metabolic syndrome and myocardial infarction [8].

To allow appropriate implementation of the breastfeeding recommendations, a knowledge of the positive effects on mother and child alone is not enough. The international Baby-Friendly Hospital Initiative, founded in 1991 by WHO and UNICEF, is working continuously to improve conditions to achieve higher breastfeeding rates and longer breastfeeding duration [9].

UNICEF data released in 2021 show that worldwide, only 44% of newborns are exclusively breastfed for the first 5 months of life. The highest prevalence is reported in South Asia, with a 57% rate of exclusive breastfeeding, in comparison with only 26% in North America [10]. According to SUKIE 2021, a study on breastfeeding behaviour and child nutrition in Austria, 97.5% of mothers stated that they had breastfed their child at some time. The breastfeeding prevalence at 6 months of age was 64.1% [11].

A prenatally established intention to breastfeed, as well as previous experience, has been shown to have a significant influence on the duration of breastfeeding. Attaching a high value to the benefits of exclusive breastfeeding is associated with a significantly longer duration. Nevertheless, only a few mothers achieve their intended goal for breastfeeding duration [12, 13]. Studies report different reasons for early cessation, such as cracked nipples, pain, or an insufficient amount of milk [14]. There is a high level of social and educational awareness of the proven benefits of breastfeeding. This might lead to intense social pressure on women and can result in feelings of guilt and inadequacy if the practice of breastfeeding does not meet expectations [15]. Only little research has been carried out on maternal satisfaction with breastfeeding, and this might also play a role [16].

The aim of this study was to analyse breastfeeding behaviour in primiparous women relative to their expectations and individual concerns. In addition, it examined the influence of social environment, prepartum knowledge and information, sociodemographic factors and pregnancy-related and birth-related factors. This information is to be used to develop measures to help women to meet the breastfeeding recommendations.

## Methods

A prospective questionnaire-based study was conducted, comprising three questionnaires at three defined survey time points. Inclusion of patients in the study and the first written interview took place during routine antepartum presentations at Kepler University Hospital – at the earliest in gestational week 34 + 0. The second questionnaire was completed during the patients’ postpartum hospitalization. The third written survey was conducted after the newborn had reached 6 months of age. The study period was set from June 2020 to April 2022. All primiparous women who delivered infants at Kepler University Hospital between 38 and 42 weeks of gestation were eligible for the final analyses. All three questionnaires had to have been completed for inclusion in the study.

Basic requirements for participation were a signed consent form and a good knowledge of German or English in order to answer the questionnaires in a meaningful way. Mothers with preterm deliveries and women with multiple pregnancies were excluded. Further exclusion criteria were prenatal fetal diagnoses or postpartum diagnoses that did not allow exclusive breastfeeding without restrictions. Women with chronic illnesses, long-term medication, or prior breast surgery limiting unrestricted breastfeeding were also excluded.

The questionnaires contained between 10 and 23 questions, most of them being single-choice or multiple-choice, about the patients’ intention to practice breastfeeding and the personal importance they attached to it, the knowledge and information they had gathered, fears, delivery mode, reasons for stopping breastfeeding, and a few open questions on sociodemographic data. Exclusive breastfeeding was defined as breastfeeding without additional supply of infant formula, except during the first few days after delivery until full lactogenesis, if necessary. The term ‘partial breastfeeding’ included supplemental feeding with infant formula and later the addition of baby food alongside breastfeeding. The English version of all three questionnaires can be found in the Additional file 1.

Statistical analysis was carried out using SPSS, version 14.0. The chi-square test or Fisher’s exact test (for fourfold contingency tables) was used for hypothesis testing, and the McNemar test was used for connected samples. The significance level was set at 5%. In some cases, dichotomizations were performed if the test criteria were not met due to the number of cases being too low.

## Results

A total of 180 primiparous women who delivered between 38 and 42 weeks of gestation at Kepler University Hospital received all three questionnaires, and the response rate was 78%. The final analyses included 140 primiparous women.

### Sociodemographic characteristics

The mean age of the participants was 30 (± 5), the youngest mother being 18 and the oldest 42 years old. Ninety per cent of the women were born in Austria; 17.9% of the respondents had a migration background (i.e., they themselves or at least one of their parents were not born in Austria). 45.7% were married and 54.3% were in a steady relationship, so no participant was a single mother. The highest completed educational qualification was at least a high-school diploma for 70.7% of the study participants, and 49.3% were university graduates. Among the respondents, 16.4% had had one or more previous pregnancies, which had ended in early abortion, late abortion, or interrupted pregnancy. Conception occurred naturally in 90.7%, and assisted reproductive techniques were used in 9.3%. Vaginal deliveries without complications occurred in 60.7% of the infants, and 17.9% of the births required a vacuum extraction. The Caesarean section rate was 21.4%.

### Duration of breastfeeding

**Fig. 1:**
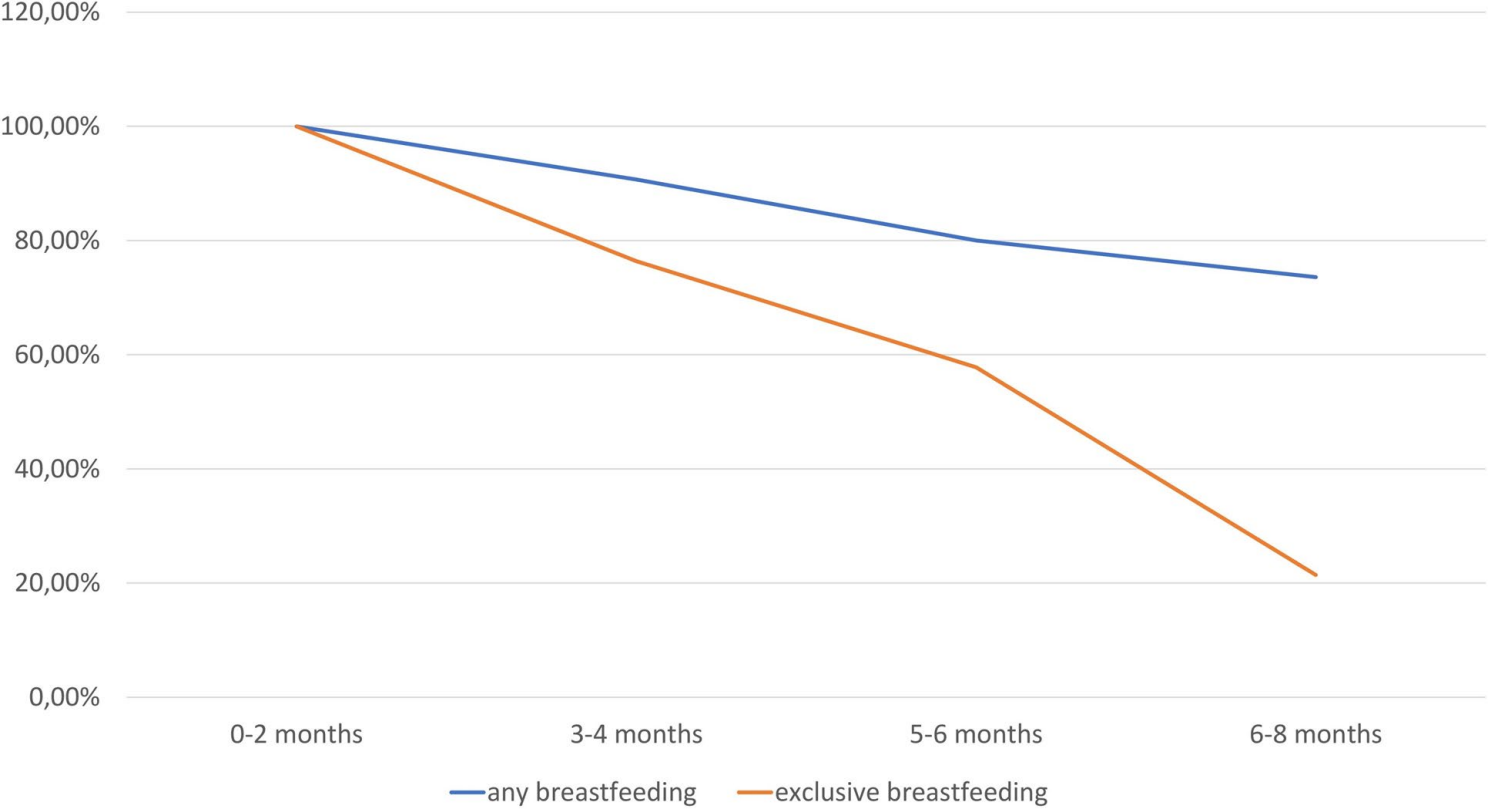
Breastfeeding prevalence. The orange line indicates the percentages (y-axis) of women who were breastfeeding exclusively, while the blue line represents the percentages of women performing any kind of breastfeeding (x-axis). It should be noted that the percentage of mothers who were breastfeeding exclusively decreased to 21% after 6 months

All of the participants (*n* = 140) stated that they had breastfed the child at some time. Fifty-eight per cent carried out breastfeeding exclusively at least until their baby had reached the age of 6 months, while 20% said they had already stopped breastfeeding at that point (Fig. 1).

**Fig. 2:**
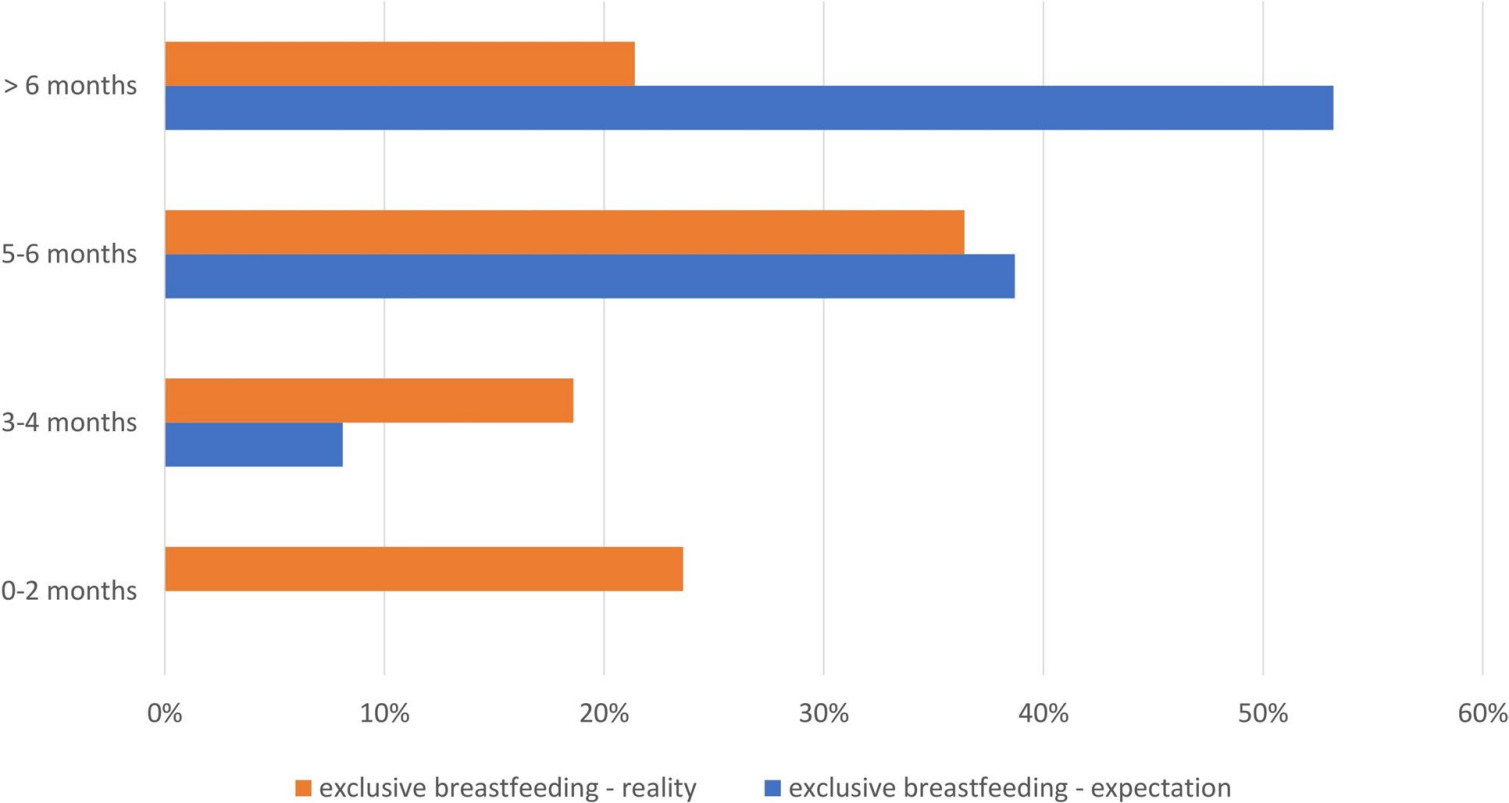
Expectations and reality. The orange bars indicate the percentages (x-axis) of women who actually breastfed exclusively for a specific time period (y-axis), while the blue bars represent their original expectations. The chart refers to an exact time period (e.g., 3–4 months) rather than the minimum breastfeeding duration and is therefore not comparable with Fig. 1. It should be noted that the breastfeeding behaviour differed significantly from the participants’ prepartum intentions

The breastfeeding behaviour differed significantly from the participants’ prepartum intentions, as 91.9% of the women had intended to breastfeed exclusively until the baby was 6 months of age. 63% of the women in this study reached their individual goal for the duration of breastfeeding. Women who planned exclusive breastfeeding for at least 6 months were significantly more likely to breastfeed exclusively only for the first 2 months (*p* = 0.048) and initiated infant formula use significantly more often (*p* = 0.012). Women who intended to breastfeed exclusively for up to 6 months were significantly more likely to cease breastfeeding within the first 6 months (*p* = 0.039) (Fig. 2).

The duration of breastfeeding was influenced by the women’s prepartum attitudes. Those who stated that they wanted to breastfeed at all costs, even though problems might occur or they might need to add infant formula, breastfed exclusively for a significantly longer period than those who stated that they wanted to at least try breastfeeding (*p* < 0.01). These two groups also differed significantly with regard to the duration of partial breastfeeding (*p* = 0.025).

The main reasons for early cessation were reported to be an insufficient quantity of milk (76.1%) and insufficient weight gain by the child (41.9%). All women who reported inadequate weight gain also reported feeling that they had an insufficient quantity of milk. Puerperal mastitis, wounded nipples, and constant pain during breastfeeding were problems in 40.4%. In 39.5% of cases, the mothers noted that the child had lost interest in the breast and had thus weaned itself. The need to abstain from alcohol and cigarettes, concerns that breastfeeding might make the breasts saggy, or the baby’s teething were not reasons for stopping breastfeeding (Table 1).

**Table 1:** Reasons for early cessation

Reasons for early cessation	Strong n (%)	Moderate n (%)	Slight n (%)	None n (%)
Insufficient quantity of milk	24 (57.1)	8 (19.0)	5 (11.9)	5 (11.9)
Too little weight gain by the baby	14 (32.6)	4 (9.3)	8 (18.6)	17 (39.5)
Baby weaned itself	12 (27.9)	5 (11.6)	8 (18.6)	18 (41.9)
Puerperal mastitis, pain, nipple cracks	9 (21.4)	8 (19.0)	8 (19.0)	17 (40.5)
Preference for feeding baby food in public	6 (14.3)	4 (9.5)	6 (14.3)	26 (61.9)
Start of feeding complementary food was a good point to stop breastfeeding	3 (7.1)	8 (19.0)	5 (11.9)	26 (61.9)
Lack of options for breastfeeding in public	3 (7.1)	4 (9.5)	5 (11.9)	30 (71.4)
Partner/friends/family should be able to feed the baby, too	2 (4.8)	4 (9.5)	12 (28.6)	24 (57.1)
Desire for greater freedom	1 (2.4)	4 (9.5)	11 (26.2)	26 (61.9)
Breastfeeding is too complicated	1 (2.4)	3 (7.3)	11 (26.8)	26 (63.4)
Breastfeeding took too much time	0 (0.0)	5 (11.9)	10 (23.8)	27 (64.3)
Breastfeeding reduced sexual desire	2 (4.8)	3 (7.1)	5 (11.9)	32 (76.2)
Desire to get more sleep again	1 (2.4)	2 (4.8)	6 (14.3)	33 (78.6)
Partner/friends/family recommended stopping breastfeeding	0 (0.0)	3 (7.1)	4 (9.5)	35 (83.3)
Worries about changes in the shape of the breast	0 (0.0)	1 (2.4)	4 (9.5)	37 (88.1)
Baby’s teething	1 (2.4)	0 (0.0)	2 (4.8)	39 (92.9)
Getting back to work	1 (2.4)	0 (0.0)	1 (2.4)	40 (95.2)
Abstaining from alcohol/cigarettes	0 (0.0)	0 (0.0)	2 (4.9)	39 (95.1)

Women who had already stopped breastfeeding their baby at the time of answering the third questionnaire, i.e. at least six months after delivery, were asked to what extent the above factors had influenced their decision. Absolute (n) and relative frequencies (%) are provided for nominal and ordinal variables. It should be noted that valid observations are used to calculate percentage values

Women who worried prepartum that the breastfed baby might not sleep through the night were significantly more likely to cease breastfeeding within the first 6 months (*p* < 0.01). The same applied to those who were concerned about not producing enough milk (*p* = 0.033). Mothers who expressed concern that there were no opportunities to breastfeed in public were significantly more likely to cease breastfeeding between 3 and 6 months (*p* < 0.01).

The reasons for early cessation did not always correlate with the participants’ expectations. Women who were initially worried about failing at breastfeeding ceased significantly more often due to limited sexual desire (*p* = 0.047). Women who were concerned about being solely responsible for feeding the baby stopped breastfeeding significantly more often in order to be able to sleep through the night again (*p* = 0.049).

### Womens’ attitude to breastfeeding

**Fig. 3:**
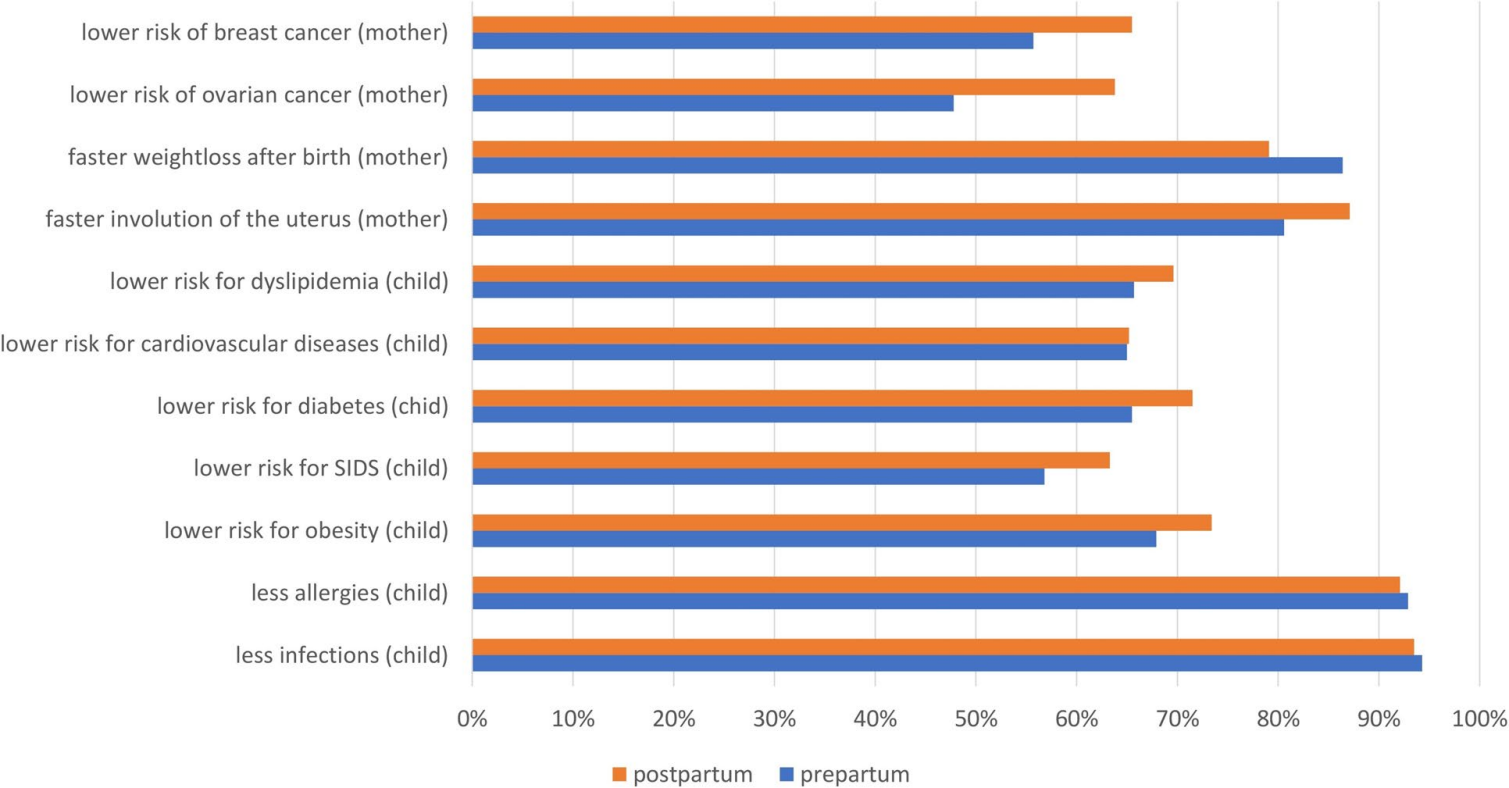
Expected benefits of breastfeeding. The women were asked to what extent (very, quite, not so much, not at all) they believed that the aspects mentioned were positively influenced by breastfeeding. The blue bars indicate in percentages (x-axis) how often the answers ‘very’ or ‘quite’ were given to each statement prepartum and the orange bars indicate the postpartum believes

Throughout all of the survey time points, the patients regarded the importance of breastfeeding for mother and child as being very important or important. The greatest benefits of breastfeeding were perceived to be the prevention of infections (94.3%) and allergies (92.9%) in the child, as well as faster involution of the uterus (80.6%) and postpartum weight loss (86.4%) (Fig. 3).

The main reasons given for breastfeeding were that breast milk is the ‘best form of nutrition’ (93.5%), the ‘most natural thing in the world’ (84.3%), and promoting mother–infant bonding (76.5%). Women who believed that breastfeeding could be done anytime and anywhere breastfed significantly longer than those who disagreed with this (*p* < 0.01). Women who believed that neither preparation nor aids were necessary for breastfeeding also had a significantly longer duration of breastfeeding (*p* = 0.012).

### Factors influencing breastfeeding

Among the study participants, 80.7% stated that they themselves had been breastfed. The friends and family members of 94.2% of them also breastfed their newborns. The women’s partners were largely in favour of breastfeeding, 15.2% of the men did not have an opinion on the subject, nor had they discussed the topic. A significant correlation between these factors and the duration of breastfeeding was not demonstrated.

Women under 30 years of age were significantly more likely to worry about being unable to breastfeed (*p* = 0.013) and not getting enough sleep (*p* = 0.040). Women over 30 were significantly more likely not to worry that the breastfed baby would not be able to sleep through the night (*p* < 0.01). The older the woman, the lower the fear of not producing enough milk (*p* < 0.01) or of the baby having too little weight gain (*p* < 0.01). The age of the study participants did not have any impact on the duration of breastfeeding.

The women’s national origin did not have any influence on the reasons they gave for breastfeeding, nor on their fears or worries. No significant differences were found with regard to the duration of breastfeeding.

Women with a higher educational level were significantly more likely to be concerned about possible breast diseases (*p* = 0.038). Women with a high-school diploma ceased breastfeeding significantly more often after more than 6 months (*p* < 0.01). Women without university entrance qualifications were significantly more likely to report that lower costs had had a positive influence on their decision to breastfeed (*p* = 0.041).

The women’s pregnancy histories and delivery-related aspects did not have any influence on the duration of breastfeeding.

Information was obtained by 74.8% of the study participants by talking to friends and family; 64.7% received appropriate advice from their midwife and 51.1% obtained additional information from the Internet. The majority of the women (54.7%) obtained the information after gestational week 22; 25.2% obtained information earlier, before gestational week 22. Information had already been obtained by 20.1% of the respondents before they became pregnant. A detailed level of knowledge before giving birth had a significant positive effect on the participants’ sense of confidence with breastfeeding (*p* = 0.028). No significant correlation was demonstrated in relation to the duration of breastfeeding. In the initial days after delivery, 87.2% of the women felt very well informed or well informed about breastfeeding; 12.1% reported a need for further information and 0.07% have not received any information at all.

The majority of the study participants, 92.1%, took advantage of postpartum midwifery care, with a positive effect on the duration of breastfeeding. They ceased breastfeeding significantly more often after more than 6 months (*p* = 0.044).

## Discussion

All participants in the study stated that they wanted to breastfeed their child, and at the time of discharge from hospital all of the children were at least being partially breastfed. Consistently with literature reports that only few women breastfeed as long as prenatally planned, 63% of the women in this study reached their goal for the duration of breastfeeding [12, 17]. Those who had strong prenatal intentions to do so breastfed significantly longer than those who reported that they at least wanted to try, but planned to stop if problems arose. Nevertheless, women who planned exclusive breastfeeding for 6 months were significantly more likely to breastfeed exclusively for only the first 2 months. Similar results have been reported in the literature; Santacruz-Salas et al. observed a five times higher odds ratio in women who intended to breastfeed exclusively for ‘as long as they could’ in comparison those who wanted to maintain it for 1, 3, or 3–6 months [13]. This might be explained by psychological discomfort, stress and fear of failure. Many women feel solely responsible for the healthy nutrition and development of the child. Due to their awareness of the benefits of breastfeeding, they may place themselves under extreme pressure and have more difficulty in coping with problems that arise [15].

The main reason for early cessation of breastfeeding in this study was reported to be an insufficient milk supply, in 75.3% of cases. However, studies have shown that in fact less than five per cent of women are biologically incapable of producing a sufficient quantity of milk [17]. The reasons for insufficient milk production are usually found to lie in poor breastfeeding techniques. The SUKIE study also reported early cessation due to insufficient milk supply in 76.1% of cases, with the difference that only primiparous women were included in the present study, whereas the proportion of primiparas in SUKIE was 52.0% [11]. Puerperal mastitis, nipple cracks, and constant pain during breastfeeding were problems in 40.4% of cases in the present study, in comparison with 32.0% of the SUKIE participants [11].

Treatment and prophylaxis is not solely the responsibility of the attending gynecologist. In addition to analgesia, anti-inflammatory therapy and antibiotic treatment if necessary, supportive measures such as frequent breastfeeding, pumping, correct positioning, breast massage and application of hot or cold compresses should be performed [18]. These measures can be communicated to the patient and checked by the midwife. Most difficulties with breastfeeding occur within the first month after delivery [14]. In Austria, health insurance covers a daily home visit by the attending midwife in the first 5 days postpartum. Moreover, an additional seven home visits or consultations can be claimed within the first 8 weeks after delivery. This service was appreciated by 92.1% of the respondents and significantly influenced the duration of breastfeeding.

The Baby-Friendly Hospital Initiative requires that all pregnant women should be informed about the benefits and practice of breastfeeding. Kepler University Hospital is no certified Baby-Friendly Hospital, but was awarded the ‘periZert’, which certifies the highest quality of care and expertise in perinatal medical care for mother and child. The WHO’s ten steps to successful breastfeeding are implemented and International Board Certified Lactation Consultants try to best support the mothers. This includes uninterrupted skin-to-skin contact after giving birth and the initiation of breastfeeding as soon as possible. Rooming-in 24 h a day is practiced and mothers are made aware of their infants’ signals for feeding and learn how to respond. Trained staff advise the mothers on the use of breast pumps and the risks of bottle-feeding or pacifiers. If the women are hospitalized during pregnancy, information on the benefits and management of breastfeeding is provided. The importance of these aspects may be underlined by the results of this study, as a detailed level of prepartum knowledge was found to have a significant impact on breastfeeding confidence. Among the study participants, 74.8% received advice from their families. Although a considerable amount of information can be passed on in this way, it is based on individual experiences that may contain negative aspects and convey uncertainty and fear. It is therefore important for patients to receive information from professionals as well. In Austria, pregnancy care includes a midwife consultation between gestational weeks 18 and 22. Among other things, the purpose of this discussion is to provide expectant mothers with basic knowledge about breastfeeding and address their fears and concerns. In the present study, 12.1% of the respondents reported needing more information in the early postpartum period. Individual counselling and support can be provided by the hospital staff during the initial days of postpartum care in hospital.

Younger women in particular benefit from detailed counselling and positive feedback. Women under 30 years of age were significantly more likely to worry about failing in breastfeeding by not providing enough milk, leading to insufficient weight gain by the baby. No effect of maternal age on the duration of breastfeeding was observed in the present study. The literature data on the topic are inconsistent. In a prospective observational study, Colombo et al. concluded that older women breastfeed less frequently, and the same finding was also reported by Kitano et al. [19, 20]. In other studies, young maternal age was associated with a shorter duration of breastfeeding [21, 22].

In the present study, the women’s national origins had no effect on the reasons given for breastfeeding, nor on their fears and worries. Nor were any significant differences observed in relation to the duration of breastfeeding. At this point, however, it should be mentioned that the composition of the study sample does not correspond to the actual population distribution in Austria, and this aspect can therefore only be assessed to a limited extent [23]. Study participants needed a good knowledge of German or English to answer the questionnaires in a meaningful way. Therefore, many women with migration background who delivered between 2020 and 2022 but did not meet these criteria could not be included in the study.

Due to the lack of a control group of single mothers, no conclusions can be drawn about the influence of partnership status on breastfeeding behaviour and duration. It has been reported in the literature that partners’ attitudes and support positively influence the initiation of breastfeeding and the duration of exclusive breastfeeding [24, 25]. Living with a partner also is associated with higher levels of maternal satisfaction with breastfeeding [16]. In this study, 15.2% of the men did not have an opinion on the subject of breastfeeding, nor had they discussed the topic with their partners. To improve breastfeeding prevalence, men should specifically be involved in the breastfeeding process and in prepartum discussions about possible difficulties to be expected and how to deal with them.

High prevalence and long duration of breastfeeding are associated with a high educational level. This has already been demonstrated in multiple studies and applies to low-income, middle-income and high-income countries [19, 26, 27]. The present study also found that women with university entrance qualifications or higher educational status were significantly more likely to cease breastfeeding only after more than 6 months. It should be noted that the study population differs from the general maternal population in Austria, in which only 42.4% successfully graduate from high school, in comparison with a university entrance qualification rate of 70.7% in the study population [28].

In contrast to the findings of other studies, the mode of birth was not found to have any influence on breastfeeding behaviour and duration. The small sample size of 140 women is a limiting factor. The main reason for less initiation of breastfeeding and continuation after Caesarean section has been cited in various studies as a disruption of the infant–mother bond [29, 30]. At Kepler University Hospital, Caesarean sections are usually performed with spinal anaesthesia so that skin-to-skin contact can be provided as early as possible. Skin-to-skin contact has been shown to promote oxytocin release, which positively affects milk production and reduces stress [31]. Initial attempts at breastfeeding can sometimes already be made in the operating room. During the first few days after delivery, women are supervised by trained lactation nurses and midwives as needed, to help them find comfortable breastfeeding positions after surgery and to resolve breastfeeding difficulties before discharge from the hospital.

Interestingly, this study was conducted during the COVID-19 pandemic. The participants’ infection status was not evaluated, although it could of course also have been an influencing factor. During the same period, multiple research projects on the impact of COVID-19 on pregnancy and outcome were conducted at Kepler University Hospital [32]. Among SARS-CoV-2–positive women, breastfeeding rates of 100% were still observed. Measures such as rooming-in or breastfeeding counselling were given a high priority even among infected women, so that they were supported in meeting their breastfeeding goals.

## Conclusions

There is a widespread desire among pregnant women to breastfeed and comply with WHO recommendations, and women are aware of the benefits of breastfeeding. A detailed level of prepartum knowledge, a high educational level and postpartum midwifery care have a positive effect on the initiation and continuation of breastfeeding. To improve breastfeeding behaviour, an additional midwifery consultation during pregnancy with a focus on breastfeeding, in the presence of the expectant father, could be considered. Low-threshold access to information and the communication of clear, easy-to-understand and consistent messages are needed. There should be a greater focus on breastfeeding follow-up, and practical structures for long-term support should be created. In addition, the social acceptance of breastfeeding should be improved. This could eliminate some of the fears, problems and concerns that women expressed, resulting in a longer duration of breastfeeding.

### Supplementary Information

**Additional file 1. **

## Data Availability

The datasets used and/or analysed during the present study are available from the corresponding author on reasonable request.

## Abbreviations

COVID-19: Coronavirus disease 2019
SIDS: Sudden infant death syndrome
SPSS: Statistical Package for the Social Sciences
SUKIE: *Säuglings- und Kinderernährung* (Study on Breastfeeding Behaviour and Child Nutrition in Austria)
UNICEF: United Nations International Children’s Emergency Fund
WHO: World Health Organisation
