# Supplementary material for: Breastfeeding in primiparous women – expectations and reality: a prospective questionnaire survey
Source: BMC Pregnancy Childbirth. 2023 Sep 9;23:654. doi: 10.1186/s12884-023-05971-1 (PMC10493027; doi:10.1186/s12884-023-05971-1)
Supplement: Supplementary file 1 — Additional file 1. [file 12884_2023_5971_MOESM1_ESM.zip › Questionnaire 1.pdf]

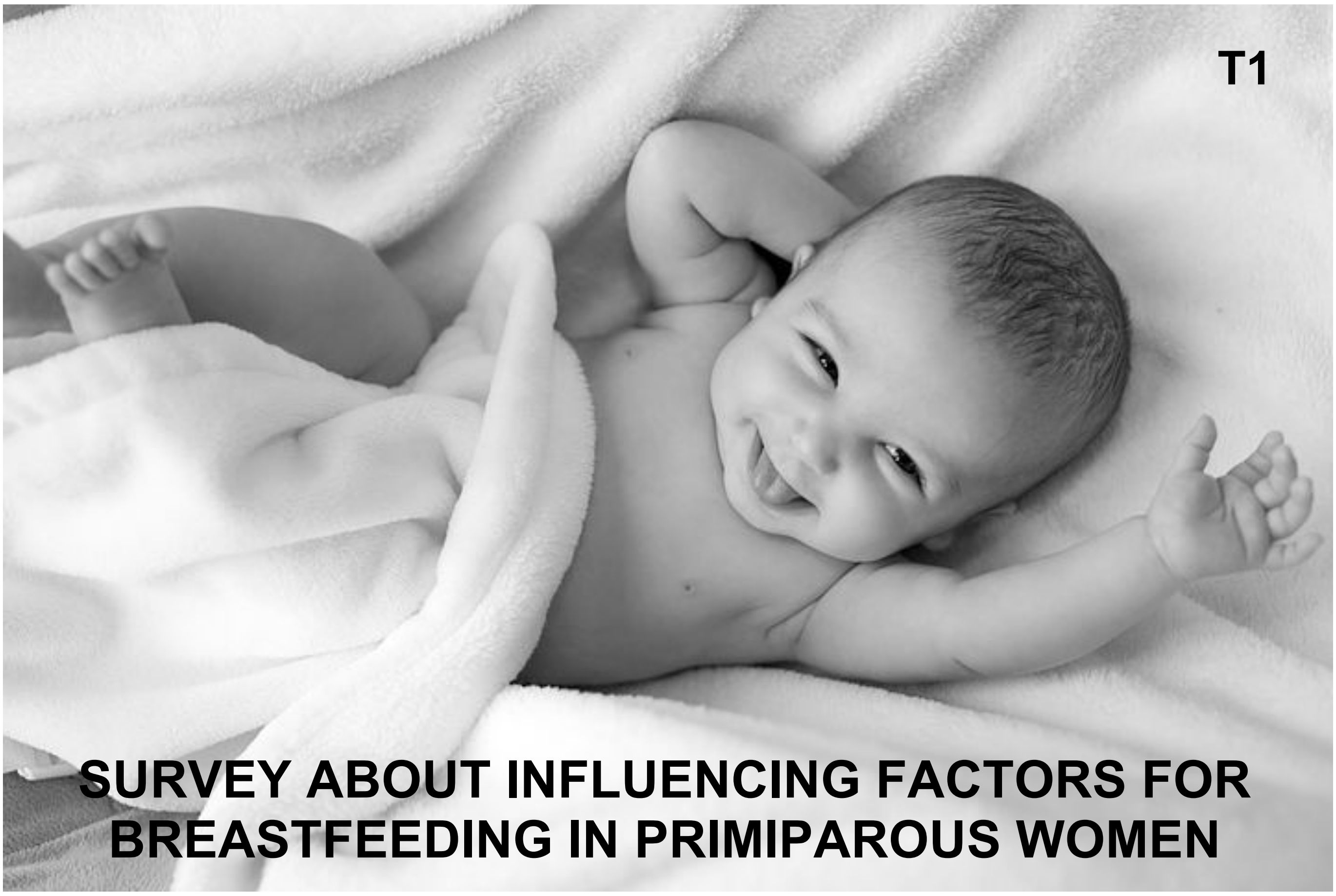

T1

SURVEY ABOUT INFLUENCING FACTORS FOR BREASTFEEDING IN PRIMIPAROUS WOMEN

Dear future mother,

please read the questionnaire point by point and choose your appropriate answer by marking it with “X” in the right box or fill in the appropriate answer in words and numbers in the according field.

This survey is anonymous. To make sure that the evaluation can be performed anonymous, we need a code to connect all your up following questionnaires.

You create the code by filling in the letters and numbers as explained:

Example: Mary Smith, born in 01.01.1990 creates the code: **m a s m 9 0**

☐	☐	☐	☐	☐	☐
First letter of your first name	Second letter of your first name	First letter of your last name	Second letter of your last name	Third letter of your birth year	Fourth letter of your birth year

Frau Katharina Hrauda

Please hand back your completed questionnaire to the staff of the obstetrical clinic before you leave.

Thank you for your participation!

Your Studyteam
Dr. Katharina Hrauda & Mag. Daniela Fritz

Kepler Universitäts Klinikum
fh GESUNDHEITS BERUFE OÖ
JKU JOHANNES KEPLER UNIVERSITÄT LINZ

1 How do you estimate your current knowledge for breastfeeding successfully? (choose only 1 answer)

I feel very well informed und know exactly what to do after the birth of my child for breastfeeding successfully.	☐
I feel well informed and know essentially what to do after the birth of my child for breastfeeding successfully.	☐
I don’t feel well informed and would need further information for breastfeeding my child after the birth successfully.	☐
I absolutely don’t feel well informed and don’t really know what to do after the birth of my child for breastfeeding successfully.	☐
I am not well informed but also don’t need further information about breastfeeding.	☐

2 Which of the following answers about breastfeeding is the most appropriate for you? (choose only 1 answer)

I definitely want to breastfeed, even if there are any difficulties.	☐
I definitely want to breastfeed, even if additional baby food is needed.	☐
I definitely want to try breastfeeding, but if there are any difficulties, I want to stop it and wean the baby.	☐
Maybe I will try to breastfeed my baby.	☐
I rather don’t want to breastfeed, but I didn’t come to a definite decision yet.→ **please continue with question 7**	☐
I am sure that I don’t want to breastfeed, because I’m not interested in it. → **please continue with question 7**	☐

3 For how long do you plan to breastfeed you baby? (1 answer per line possible)

☐ 0-2 month breastfeeding	☐ 3-4 month breastfeeding	☐ 5-6 month breastfeeding	☐ > 6 month breastfeeding
☐ 0-2 month breastfeeding and adding baby food	☐ 3-4 month breastfeeding and adding baby food	☐ 5-6 month breastfeeding and adding baby food	☐ > 6 month breastfeeding and adding baby food

4 Which answer is the most appropriate for you? (choose only 1 answer)

I am sure that I’m able to breastfeed.	☐
I sometimes have doubts if I will be able to breastfeed.	☐
I often have doubts if I will be able to breastfeed.	☐
I am afraid that I will not be able to breastfeed.	☐

5 How appropriate are the following statements for you and how did they influence your decision to breastfeed? (choose only 1 answer in a line)

	Very	Quite	Not so much	Not at all
For me, breastfeeding is the most natural behavior in the world	☐	☐	☐	☐
Breast milk is the best for my baby	☐	☐	☐	☐
Breastfeeding is important for an unique mother-child-bonding	☐	☐	☐	☐
The feeling that I can feed my baby as a mother all by myself without any help of others	☐	☐	☐	☐
Breastfeeding is available always and everywhere	☐	☐	☐	☐

Frau Katharina Hrauda

	Very	Quite	Not so much	Not at all
No preparation or device is needed for feeding my baby	☐	☐	☐	☐
Breastfeeding is cheaper than baby food	☐	☐	☐	☐
Breastfeeding is recommended by experts/friends/family	☐	☐	☐	☐

6 **You decided to breastfeed. How strong are your worries about …?** (choose only 1 answer in a line)

	Very strong	Quite strong	Not so strong	No worries at all
… getting back to work again	☐	☐	☐	☐
… failing at breastfeeding	☐	☐	☐	☐
… being the only person responsible for your baby's nutrition	☐	☐	☐	☐
…. reduced desire for sexuality	☐	☐	☐	☐
… pain caused by breastfeeding	☐	☐	☐	☐
… possible diseases of the breast (e.g. infections of the breast, lesions on nipples)	☐	☐	☐	☐
… changes of the shape of your breast	☐	☐	☐	☐
… your baby sleeping for short periods of time	☐	☐	☐	☐
… getting less sleep	☐	☐	☐	☐
… handling abstinence for alcohol, cigarettes etc.	☐	☐	☐	☐
… having to less milk	☐	☐	☐	☐
… little weight gain of your baby	☐	☐	☐	☐
… missing options for breastfeeding in public	☐	☐	☐	☐
… people's negative reactions while breastfeeding in public	☐	☐	☐	☐

→ **Please continue with question 8**

7 **You decided against breastfeeding. How important have the following aspects been for you decision-making?** (choose only 1 answer in a line)

	Very strong	Quite strong	Not so strong	No worries at all
Worries about having too less milk	☐	☐	☐	☐
Worries about little weight gain of your baby	☐	☐	☐	☐
Worries about failing at breastfeeding	☐	☐	☐	☐
Worries about being the only person responsible for your baby's nutrition	☐	☐	☐	☐
It's easier to take care of the baby for others beside the mother, if the baby is fed with baby food	☐	☐	☐	☐
Missing options for breastfeeding in public	☐	☐	☐	☐
People's negative reactions while you are breastfeeding in public	☐	☐	☐	☐

Frau Katharina Hrauda

	Very strong	Quite strong	Not so strong	No worries at all
Shame while breastfeeding in public	☐	☐	☐	☐
Easy handling of baby food in the daily routine	☐	☐	☐	☐
It's easier to get back to work early after birth	☐	☐	☐	☐
Babys fed with baby food need less meals per day	☐	☐	☐	☐
Worries about reduced desire for sexuality	☐	☐	☐	☐
Worries about pain caused by breastfeeding	☐	☐	☐	☐
Worries about possible diseases of the breast (e.g. infections of the breast, lesions on nipples)	☐	☐	☐	☐
Worries about changes of the shape of your breast	☐	☐	☐	☐
Babys fed with baby food tend to sleep for longer periods	☐	☐	☐	☐
You are rather getting more sleep	☐	☐	☐	☐
You don't have to abstain from alcohol, cigarettes etc.	☐	☐	☐	☐
Family and friends recommended to not breastfeed	☐	☐	☐	☐

8 How do you estimate the importance of breastfeeding for mother and child generally?
(choose only 1 answer in a line)

	Very	Quite	Not so much	Not at all
For the mother	☐	☐	☐	☐
For the child	☐	☐	☐	☐

9 What do you think, how much can the following aspects be influenced by breastfeeding in a positive way? (choose only 1 answer in a line)

	Very	Quite	Not so much	Not at all
Children get less infections	☐	☐	☐	☐
Children get less allergies	☐	☐	☐	☐
Children are at lower risk for obesity	☐	☐	☐	☐
Children are at lower risk for sudden infant death	☐	☐	☐	☐
Children are at lower risk for diabetes	☐	☐	☐	☐
Children are at lower risk for cardiovascular diseases	☐	☐	☐	☐
Children are at lower risk for fat metabolism disorders	☐	☐	☐	☐
Quick postpartum uterine regression (mother)	☐	☐	☐	☐
Quick postpartum weight loss (mother)	☐	☐	☐	☐
Reduction of risk for developing ovarian cancer (mother)	☐	☐	☐	☐
Reduction of risk for developing breast cancer (mother)	☐	☐	☐	☐

Frau Katharina Hrauda

10 In which ways did you get your information about breastfeeding yet?
(choose as many answers as you like)

☐ Friends and/or family	☐ Gynecologist	☐ Familydoctor or Pediatrician
☐ Midwife	☐ Prenatal classes	☐ Internet
☐ Books	☐ Other sources of information	☐ not at all → **please continue with question 12**

11 When was the first time that you gathered information about breastfeeding? (choose only 1 answer)

☐ Before I got pregnant	☐ Until the 22nd week of pregnancy	☐ After the 22nd week of pregnancy

12 Did your own mother breastfeed you? No matter for how long. (choose only 1 answer)

☐ Yes	☐ No	☐ I don't know

13 In your circle of friends and family, is breastfeeding for newborns predominant?
(choose only 1 answer)

☐ Yes	☐ No

14 What does your partner think about breastfeeding? (choose only 1 answer)

He/she thinks that breastfeeding is important	☐
He/she doesn't have a clear opinion about breastfeeding	☐
He/she is against breastfeeding	☐
I have not talked to him/her about breastfeeding yet	☐
Right now, I'm not in a relationship	☐

15 What is applicable for your pregnancy? (choose only 1 answer in a line)

	yes	no	
This is my first pregnancy	☐	☐	
I already had an abortion until the 12th week of pregnancy	☐	☐	If yes, how many: ___________
I already had an abortion after the 12th week of pregnancy or a stillbirth	☐	☐	If yes, how many: ___________
I already had an induced abortion	☐	☐	If yes, how many: ___________
I already had an ectopic pregnancy	☐	☐	If yes, how many: ___________

16 How did you get pregnant? (choose only 1 answer)

Natural, spontanous	☐
Medical assisted	☐

Frau Katharina Hrauda

17 How old are you?

Age in years: ___________

18 Have you been born in Austria? Have your parents been born in Austria?
(choose only 1 answer in a line)

	Yes, in Austria	No, in (please fill in the country)	I don't know
Yourself	☐	______________________________	☐
Your father	☐	______________________________	☐
Your mother	☐	______________________________	☐

19 Which language do you use at home? (choose only 1 answer)

☐ German	☐ Albanian	☐ Arabic	☐ Bosnian
☐ Chinese	☐ English	☐ French	☐ Italian
☐ Croatian	☐ Kurdish	☐ Mazedonian	☐ Persian
☐ Polish	☐ Portuguese	☐ Romanes	☐ Romanian
☐ Russian	☐ Serbian	☐ Slovakian	☐ Slowenian
☐ Spanic	☐ Czeck	☐ Turkish	☐ Hungarian
☐ other language:	____________________		

20 Are you part of a religious group? (choose only 1 answer)

☐ catholic	☐ orthodox	☐ evangelical	☐ other christian church
☐ islamic	☐ buddhistic	☐ other religion	☐ no religious denomination

21 Are you…? (choose only 1 answer)

☐ single	☐ in a relationship	☐ married
☐ divorced	☐ widowed	

22 What is your highest qualification? (choose only 1 answer)

☐ Compulsory school	☐ Apprenticeship	☐ Intermediate technical or vocational school
☐ secondary school (Matura)	☐ University degree / college	

Frau Katharina Hrauda

[illegible]3 Before your maternity protection time you were …? (choose only 1 answer)

☐ Worker	☐ Employee	☐ Official / public service	☐ Self-employed
☐ Farmer / forestry	☐ Working in the household	☐ In training	☐ Job searching

THANKS FOR YOUR PARTICIPATION AND ALL THE BEST!
