# Supplementary material for: Breastfeeding in primiparous women – expectations and reality: a prospective questionnaire survey
Source: BMC Pregnancy Childbirth. 2023 Sep 9;23:654. doi: 10.1186/s12884-023-05971-1 (PMC10493027; doi:10.1186/s12884-023-05971-1)
Supplement: Supplementary file 1 — Additional file 1. [file 12884_2023_5971_MOESM1_ESM.zip › Questionnaire 3.pdf]

Version 1.2, 16.05.2020

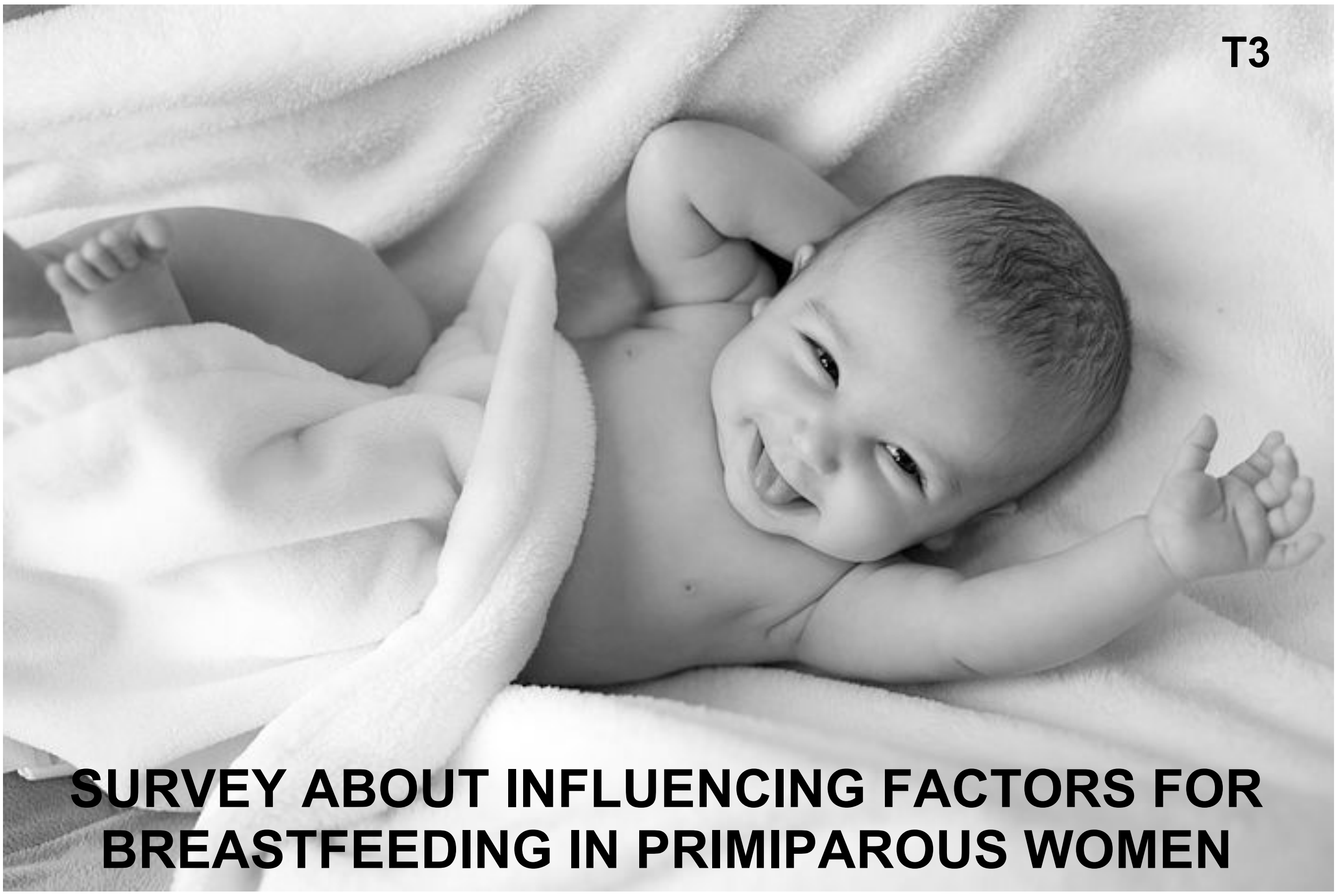

T3

SURVEY ABOUT INFLUENCING FACTORS FOR BREASTFEEDING IN PRIMIPAROUS WOMEN

Dear mother,

please read the questionnaire point by point and choose your appropriate answer by marking it with “X” in the right box or fill in the appropriate answer in words and numbers in the according field.

This survey is anonymous. To make sure that the evaluation can be performed anonymous, we need a code to connect all your up following questionnaires.

You create the code by filling in the letters and numbers as explained:

Example: Mary Smith, born in 01.01.1990 creates the code: **m a s m 9 0**

First letter of your first name	Second letter of your first name	First letter of your last name	Second letter of your last name	Third letter of your birth year	Fourth letter of your birth year

Frau Katharina Hrauda

Please send back the completed questionnaire by post. Therefore, you find a prepaid envelope added.

Thank you for your participation!

[illegible]ur Studyteam
[illegible] Katharina Hrauda & Mag. Daniela Fritz

Kepler Universitäts Klinikum
GESUNDHEITS BERUFE OÖ
JOHANNES KEPLER UNIVERSITÄT LINZ

1 **For how long did you breastfeed your baby exclusively?** (choose only 1 answer)

☐ 0-2 months ☐ 3-4 months ☐ 5-6 months ☐ > 6 months **→ continue with question 6**

2 **At which time did you start to add baby food beside breastfeeding?** (choose only 1 answer)

☐ not yet ☐ after 0-2 months ☐ after 3-4 months ☐ after 5-6 months ☐ after > 6 months

3 **Did you stop breastfeeding in the meantime to wean your baby?** (choose only 1 answer)

☐ yes, after 0-2 months ☐ yes, after 3-4 months ☐ yes, after 5-6 months ☐ yes, after > 6 months

☐ no **→ continue with question 6**

4 **You already stopped breastfeeding. How much did the following factors influence your decision to wean your baby?** (choose 1 answer in a line)

	Very	Quite	Not so much	Not at all
Too less milk	☐	☐	☐	☐
Too little weight gain of your baby	☐	☐	☐	☐
Your baby weaned itself, it lost its interest in breastfeeding	☐	☐	☐	☐
Breastfeeding took too much time	☐	☐	☐	☐
Breastfeeding was too complicated	☐	☐	☐	☐
Also your partner or friends/family should have the possibility to take care of the baby by feeding it with baby food	☐	☐	☐	☐
Desire for more liberty, when you don’t have to be around all time	☐	☐	☐	☐
Getting back to work again	☐	☐	☐	☐
Your partner, friends or family recommended to stop breastfeeding	☐	☐	☐	☐
You didn’t want to abstain from alcohol, cigarettes etc.	☐	☐	☐	☐
You had infections of the breast, lesions on nipples or pain caused by breastfeeding	☐	☐	☐	☐
Breastfeeding reduced your desire for sexuality	☐	☐	☐	☐
You worried about changes of the shape of your breast	☐	☐	☐	☐
You wanted to get more sleep again	☐	☐	☐	☐
Missing options for breastfeeding in public	☐	☐	☐	☐
You prefer to feed your baby with baby food in public	☐	☐	☐	☐
You were bothered by developing teeth of your baby	☐	☐	☐	☐
When you started to feed your baby with complementary food, it was a good point to wean the baby	☐	☐	☐	☐
You had to take some medicine that was incompatible with breastfeeding	☐	☐	☐	☐

Frau Katharina Hrauda

5 **Under certain conditions, could you imagine to breastfeed for a longer period of time?** (choose only 1 answer)

Yes, I would have liked to breastfeed for a longer time.	☐
Rather yes, under certain conditions I would have continued to breastfeed for a longer time.	☐
Rather no, the decision to wean my baby was fine for me.	☐
No, weaning my baby was just at the right time for me.	☐

6 **How do you estimate the importance of breastfeeding for mother and child generally?** (choose only 1 answer in a line)

	Very	Quite	Not so much	Not at all
For the mother	☐	☐	☐	☐
For the child	☐	☐	☐	☐

7 **What do you think, how much can the following aspects be influenced by breastfeeding in a positive way?** (choose only 1 answer in a line)

	Very	Quite	Not so much	Not at all
Children get less infections	☐	☐	☐	☐
Children get less allergies	☐	☐	☐	☐
Children are at lower risk for obesity	☐	☐	☐	☐
Children are at lower risk for sudden infant death	☐	☐	☐	☐
Children are at lower risk for diabetes	☐	☐	☐	☐
Children are at lower risk for cardiovascular diseases	☐	☐	☐	☐
Children are at lower risk for fat metabolism disorders	☐	☐	☐	☐
Quick postpartum uterine regression (mother)	☐	☐	☐	☐
Quick postpartum weight loss (mother)	☐	☐	☐	☐
Reduction of risk for developing ovarian cancer (mother)	☐	☐	☐	☐
Reduction of risk for developing breast cancer (mother)	☐	☐	☐	☐

8 **What does your partner think about breastfeeding?** (choose only 1 answer)

He/she thinks that breastfeeding is important	☐
He/she doesn't have a clear opinion about breastfeeding	☐
He/she is against breastfeeding	☐
[illegible] have not talked to him/her about breastfeeding yet	☐
[illegible]ght now, I'm not in a relationship	☐

Frau Katharina Hrauda

9 Did you have any assistance from a midwife at home after your discharge from hospital? (choose only 1 answer)

☐ Yes ☐ No

10 How old is your child in weeks?

Weeks: ___________

THANKS FOR YOUR PARTICIPATION! WE WISH YOU AND YOUR FAMILY ALL THE BEST!

Frau Katharina Hrauda
